# Effect of the Position of the Boundary Rivets on the Quality of Riveted Single Strap Butt Joints

**DOI:** 10.3390/ma14185127

**Published:** 2021-09-07

**Authors:** Anan Zhao, Yongliang Zhang, Chunrun Zhu, Zhengwei Zhong, Yunbo Bi

**Affiliations:** 1 State Key Laboratory of Fluid Power and Mechatronic System, College of Mechanical Engineering, Zhejiang University, Hangzhou 310027, China; swuoip@163.com (A.Z.); zhangyl009@avic.com (Y.Z.); 2 Key Laboratory of Advanced Manufacturing Technology of Zhejiang Province, College of Mechanical Engineering, Zhejiang University, Hangzhou 310027, China; zjuzcr@zju.edu.cn (C.Z.); 21825217@zju.edu.cn (Z.Z.); 3 Aviation Industry Corporation of China Xi’an Aircraft Industry (Group) Limited Company, Xi’an 710089, China; 4 Aviation Industry Corporation of China Shenyang Aircraft Corporation, Shenyang 110850, China

**Keywords:** single strap butt joints, rivet irregular layout, nail load distribution, finite element simulation

## Abstract

Riveting is widely used in aircraft manufacturing. The strap butt joint is often used in the aircraft’s main bearing area such as the aircraft docking area. The connection quality affects the reliability and safety of the aircraft directly. To study the effect of the rivet position on the connection quality of the strap butt joints, this paper analyzed the distribution of stress around the rivet hole at different positions by the finite element method, and then further analyzed the influence of the different rivet layouts on the connection quality of the strap butt joints by experiments. The static load tensile failure test of the joints was carried out, and the obtained tensile strength and failure mode of the strap butt joints showed that the main static tensile failure form of the single strap butt joint is that the whole rivets is sheared and the connecting sheets are separated. By changing the layout of different rivets, the connection strength can be maximized by reducing the outer row spacing (ORSD) of rivets. The results can be used for reference in the design of the riveting structure of aircraft panels.

## 1. Introduction

Due to the constraints of design, manufacturing, assembly and other factors, aircraft fuselages usually need to be segmented. The number and location of the fuselage sections vary depending on the type and passenger capacity. Aircraft fuselage can usually be divided into nose, front fuselage, middle fuselage, rear fuselage and the tail section. Under the premise of satisfying the structural integrity requirements of the aircraft, the connecting structure must not only ensure the reliable transmission of the load, but also ensure the smoothness of the fuselage surface and a good aerodynamic shape. At the same time, it must also take the requirements of sealing into account. Therefore, most of the connection schemes adopt butt joints, and the main butt joints include the strap plate butt joint, sleeve-type butt joint and joint-type butt joint. As a docking connection method, the strap plate connection is widely used in the aircraft assembly of the fuselage and wings [1]. 

**Figure 1:**
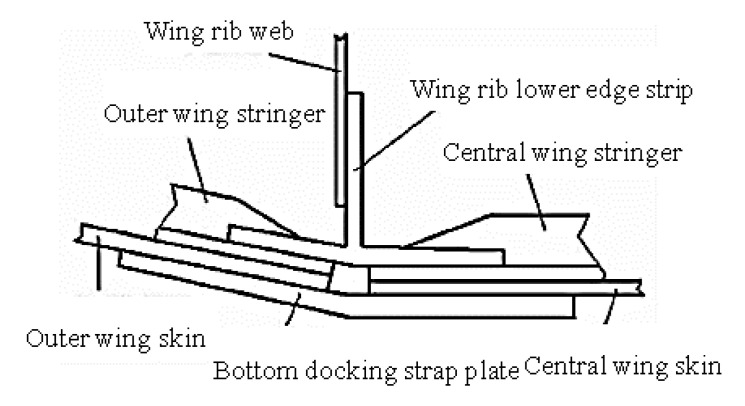
Schematic diagram of the docking of the lower panel of a typical metal wing.

Wing sheets are usually composed of several sheets. The sheets are generally connected by lap joints or butt joints. The lap joint scheme has a small connection area and uses fewer connectors, but the lap wall may have a sinking problem, which causes the structural stress to be concentrated and affects the fatigue performance of the wing. Due to the use of strap plates and more connectors, the docking solution adds extra weight. However, the butt connection can effectively avoid sinking, and the fatigue performance is better. In the butt joint scheme, the high-stress area generally appears on the strap plate, the repair of the strap plate is relatively simple, and the crack can be stopped in time after the crack occurs. At present, in the structural design of civil airliners, the connection of the lower wing panels adopts the docking form shown in Figure 1. 

**Figure 2:**
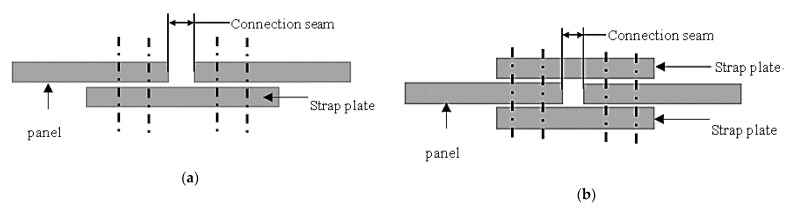
Schematic diagram of typical strap plate connection structures (**a**) single strap and (**b**) double strap.

Strap plate connection is also used in the structure of aircraft skin connection. The structure of the fuselage skin connection is related to factors such as the number of fasteners, the location of the long stringers, and the use of strap plates. Figure 2 lists two structural forms of the fuselage longitudinal skin connection. The structure of Figure 2a is a single-strip plate lap joint, and the structure of Figure 2b is a double-strip plate lap joint.

For the connection of sheets in aerospace structures, especially the riveting of aircraft fuselage and wing sheet structures, the process research focuses on the fatigue life, strength and residual stress of thin-walled parts caused by riveting. Stress–strain analysis is an important basis for research on riveting technology. Li et al. [2] studied the effects of clearance fit, friction coefficient, and corrosion expansion on the stress state of three-row riveted laps. The study showed that the size of clearance fit affects the maximum stress position near the top fastener hole, and the larger friction coefficient increases the contact stress and friction stress near the hole, and corrosion expansion significantly increases the stress amplitude near the hole. Cabrera et al. [3] conducted finite element analysis on basic load conditions and riveted parts of different materials, adjusted them by the least square method, and obtained polynomial expressions of stress distribution under all conditions. Eslami et al. [4] proposed a residual stress model to predict the residual stress distribution after riveting. The model is used to predict the change of the residual stress on the riveted surface, with the changing parameters being the riveted joint height, hole diameter, material properties, etc. and is verified by experiments. Atre et al. [5] studied the distribution of stress and strain around the riveting hole by finite element simulation, observed the deflection residual stress distribution during the riveting process and the residual stress under the pressurized airframe, and studied the impact of interference, sealing and drill cuttings on the stress state. 

Connection strength and fatigue life are important factors for the quality evaluation of connectors. Yu et al. [6] conducted a numerical simulation and experimental research on different riveting sequences, riveting methods and rivet spacings of single-row and three-row lap joint rivets, and established the fatigue life prediction model of a multi-rivet lap joint considering the coupling effect of residual stress and cyclic load. Aman et al. [7] studied the best riveting sequence by the finite element simulation method to reduce residual stress and improve the quality of riveted joints. Rans et al. [8] studied the influence of the pressure riveting force and the residual stress generated by its interference fit on the fatigue cracks of the riveted parts, and the results showed that a larger pressure riveting force can effectively reduce the rate of crack growth. Manes et al. [9] studied the influence of pressure riveting force, rivet length, and hole diameter tolerances on the riveting quality during the riveting process, and they found that the pressure riveting force had the greatest influence on riveting fatigue life. Naarayan et al. [10] studied the influence of the uneven distribution of rivet load on the fatigue life of riveted parts. The study found that the rivet load distribution in the composite material and metal lap joint is asymmetrical, and the uneven distribution causes some rivets to bear a higher load, so cracks and damage are more likely to occur. For the multi-rivet connection structure, the distribution of the nail load is a factor that needs to be considered in the design. Tate et al. [11] proposed the spring mass point model for the first time and analyzed the pin loading of the isotropic plate. Nelson et al. [12] optimized the spring mass point model and calculated the nail load of the single-lap structure. McCarthy et al. [13] considered the situation with gaps in nail holes.

When the aircraft sheet or skin adopts a strap plate connection scheme, multiple rows of rivets are generally used for connection, and the arrangement of the rivets is a multi-row and multi-column pattern. Except for the edges of the multi-row and multi-column connection area, the force between the rivets in each row, the rivet bearing ratio and the stress–strain state of the sheet are similar when the connector is loaded, so this paper adopts the arrangement of three rows of rivets as the research object. Multi-row rivet connection causes the uneven loading of the rivets, resulting in a large load on the rivets at both ends, which becomes a weak link that restricts the performance of the connector. In order to avoid the “peak effect” of multiple rows of rivets and improve the connection quality of the strap butt joints, the position of rivets at the edge is changed in the practical engineering to improve the quality of the connectors, but the influence law of changing the position of the rivets on the quality of the connectors is still unclear. Position changes are mostly carried out according to empirical formulas, and the internal influence mechanism is not clear. Therefore, on the basis of numerical analysis, this paper carries out the research on the influence of the rivet position on the connection quality of the strap plate connection. With the aid of the finite element analysis software ABAQUS, a simulation model of the strap butt joints is established. The distribution of rivet load is calculated by analyzing the stress and deformation after riveting. By designing the static load tensile failure test of the strap butt joints, the failure form of the strap butt joints is analyzed, the nail load distribution experiment is carried out, and the simulation result is compared with the test result to verify the correctness of the model. By changing the position of the rivet to adjust the distribution of the rivet load and the size and distribution of the residual stress of the plate, thereby improving the connection quality and mechanical properties of the strap-plated connector, the relevant influence law of the rivet position on the connection quality is summarized.

## 2. Modeling and Simulation of Riveted Single Strap Butt Joints

### 2.1. Design of Rivets Arrangement

According to the analysis of the load transmission of multi-row and multi-column connectors, three-rivet connectors with the same rivet spacing show a rule that the bearing ratio of rivets is larger at both ends and small in the middle of one column when subjected to tensile load. Therefore, in order to improve the connection quality of the connector, this paper adjusts and compares the positions of the outer rivets of each column to obtain a more uniform load and improve the connection quality as the main goal. By studying the influence of rivet position on the nail load distribution, stress distribution and load-bearing capacity of the connector under tensile load, while observing its influence on the stress distribution of the current column of rivets, the research of the rivet position arrangement with variable spacing is realized.

**Figure 3:**
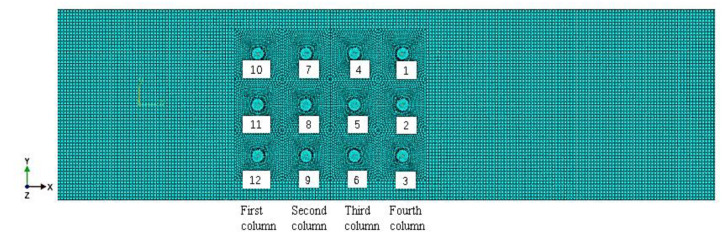
Schematic diagram of the evenly arranged rivet layout and mesh of strap butt joints.

Based on the uniformly arranged connectors shown in Figure 3, the positions of the rivets 2, 5, 8, and 11 in the middle row are kept unchanged, and the position of the outer rivets is changed. By adjusting the position of the rivets on both sides of the inner (second and third columns) or the outer (first and fourth columns), the distance between the rivets at both ends or the row spacing distance with the adjacent column of rivets is changed, and the influence of different rivet positions on the stress distribution of the connector is studied. 

**Figure 4:**
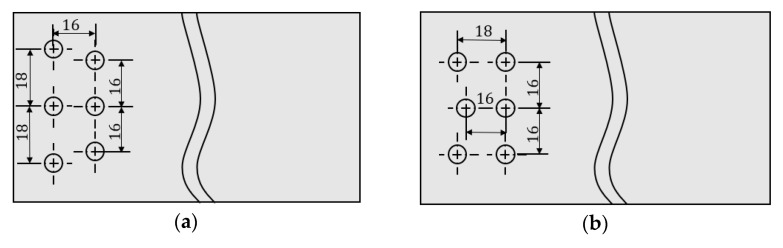

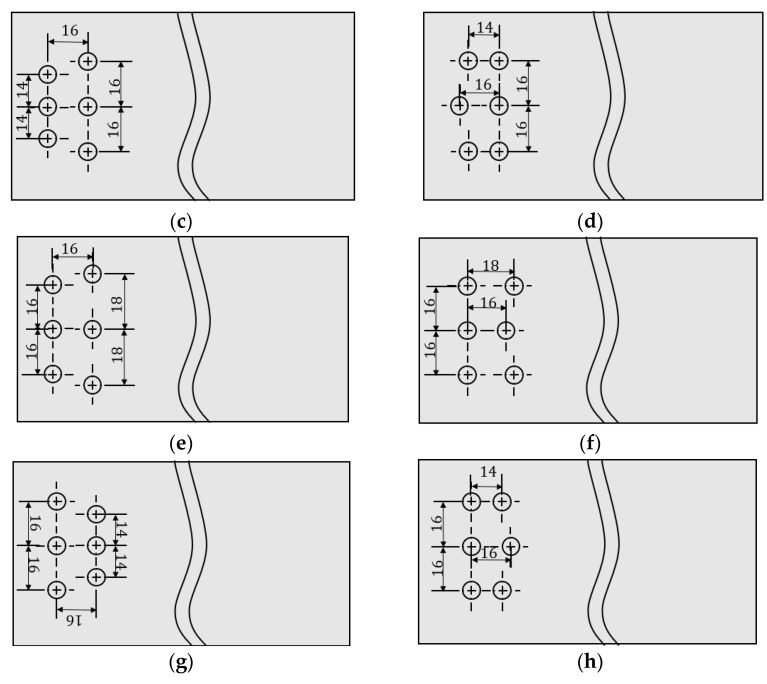
Different rivet layout design: (**a**) increase the outer rivet spacing (OSI); (**b**) increase outer rivet row spacing (ORSI); (**c**) decrease the outer rivet spacing (OSD); (**d**) decrease outer rivet row spacing (ORSD); (**e**) increase the inner rivet spacing (ISI); (**f**) increase inner rivet row spacing (IRSI); (**g**) decrease the inner rivet spacing (ISD); (**h**) decrease inner rivet row spacing (IRSD).

In order to facilitate the distinction, the arrangement of rivets in different positions is abbreviated. Among them, ‘O’ and ‘I’ mean outside or inside, the middle ‘S’ and ‘RS’ mean spacing or row spacing, and the last ‘I’ and ‘D’ means increase or decrease. According to the relevant design rules of aircraft assembly [14], the moved distance of rivets at different positions is taken as 2 mm. The change plan of rivet spacing or row spacing at each position inside and outside is shown in Figure 4.

### 2.2. Finite Element Modeling

Since the sheets undergo radial deformation after riveting, it may have a certain impact on the gap between the sheets. If the butt gap between the sheets is small, the adjacent sheets may contact and squeeze after the riveting, and consequently both sheets will be deformed. Therefore, it is more appropriate for the gap of the seam to be more than twice the deformation in actual application. In this paper, the gap of the seam is 1 mm.

**Figure 5:**
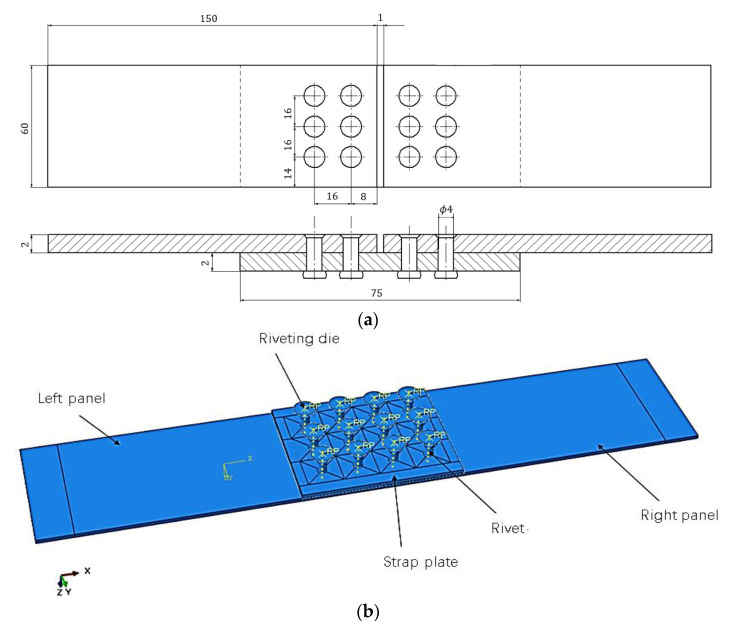
Schematic diagram of the structure of the strap butt joints: (**a**) schematic diagram of the composition and size of the strap butt joints(unit:mm); (**b**) schematic diagram of finite element model riveting assembly.

The structural parameters of the strap butt joints are shown in Figure 5a. It is composed of a left sheet, right sheet, strap plate, rivets, riveting die and other parts. Among them, the sheets and strap plate use the same AL2024-T3 material, and their thicknesses are both 2 mm. The nail hole diameter is 4.08 mm, the rivet material is AL2117-T4 and its standard grade is YSA622-100° countersunk head rivets. The detailed material parameters are as shown in Table 1. According to the actual riveting process, a finite element model of riveted parts with a strap plate, as shown in Figure 5b, is established.

According to the test data, the relationship of the stress (*σ*_true_) and strain (*ε*_true_) of the aluminum alloy in the plastic deformation stage satisfies

(1) $$\sigma_{\mathrm{true}}=C{\left(\varepsilon_{\mathrm{true}}\right)}^{m}$$

Since the riveting die has small deformation relative to other parts, it is set as a rigid body in the finite element model. The riveting analysis is a complex nonlinear contact problem, and the plastic deformation is mainly concentrated on the rivet and the periphery of the connected part during riveting. In order to improve the simulation accuracy and calculation efficiency, the mesh around the rivet hole is refined; the rivet, sheet and the strap plate mesh use C3D8R unit type.

The analysis step of the simulation is divided into two steps of riveting and stretching. During the riveting process, for the joints shown in Figure 3, rivets 1 to 12 undergo riveting in sequence. According to the division of the riveting process stages and the law of motion, the single riveting process is divided into a loading process and an unloading process, so two analysis steps are set for each rivet. Since the riveting process is a high-speed, high-strain impact load formation process, the explicit analysis module is used for simulation. The specific analysis steps are as follows: 

(1) Loading process: This starts with the movement of the riveting die at the initial position and ends when the riveting die moves to the maximum riveting distance. This stage is the main stage of the riveting process. During the loading process, the rivet is deformed under compression to form a pier head. The analysis step time of a single rivet loading process is 1 min. 

(2) Unloading process: Starting from the riveting die at the maximum riveting distance, the riveting die is set back. The riveting die is separated from the pier head and continues to retreat to a certain distance. The analysis step time of a single rivet unloading process is 1 min. 

(3) On the basis of the completion of the riveting, the joint is stretched. A shearing force is applied in the positive direction of X to the clamping part of sheet, and the tensile load F = 25 KN, which is consistent with the test. The tensile analysis step uses the force load control method; the loading time is 5 min, and the tensile load increases linearly. 

**Figure 6:**
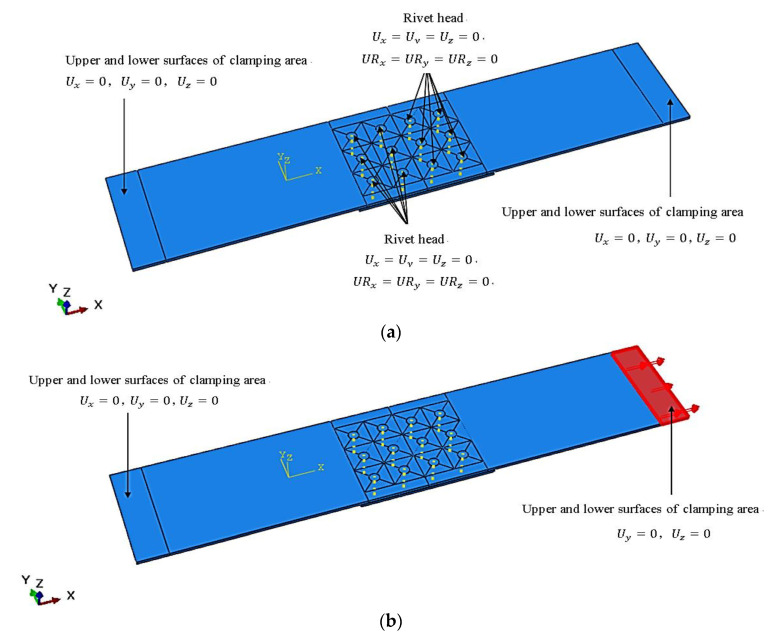
Boundary condition setting for the analysis step of the strap butt joints: (**a**) boundary condition setting of riveting step; (**b**) boundary condition setting of stretching step.

The setting of model boundary conditions and degrees of freedom is shown in Figure 6. By limiting the degree of freedom of the rivet head, it simulates the fixation of the presser foot during the riveting process. The degree of freedom of the areas is limited at both ends of sheets to simulate the clamping situation during press riveting process. During the riveting operation, the clamping area and the presser foot area are fixed at both ends. In the stretching simulation step, the clamping constraint is canceled at one end and the positive X shear force is applied instead.

The riveting process can be controlled in two ways: force load and displacement load. In this paper, the pressure riveting adopts the displacement control method. According to the principle of rivet volume conservation, the calculation formula of the pressure riveting amount can be obtained as [7]:

(2) $$S\;=\;L_{2}{\;-\;(t}_{1}{\;+\;t}_{2}{\;-\;t}_{3})\;-\;\frac{d^{2}L_{2}\;-\;d_{3}^{2}(t_{1}{\;+\;t}_{2}{\;-\;t}_{3})}{D_{p}^{2}}$$

In the formula, L_2_ is the length of the rivet rod; t_1_ is the thickness of the sheet; t_2_ is the thickness of the strap plate; t_3_ is the height of the rivet head; d is the diameter of the rivet; d_3_ is the hole diameter of the sheet; D_p_ is the diameter of the pier head after pressure riveting. Substituting the corresponding parameters into the calculation, the downward pressure of the riveting die can be obtained as 3.71 mm.

According to the analysis of the above riveting process, the main contact pairs in the model are as follows: (1) the contact between the bottom surface of the riveting die and the side face of the rivet rod; (2) the contact between the cylindrical surface of the rivet and the wall of the sheet siding hole; (3) the contact between the cylindrical surface and the countersunk surface of the rivet; (4) the contact between the wall of the sheet hole and the countersunk hole; (5) the contact between the sheets and the strap plate; (6) the contact between the pier head surface and the sheet surface after the rivet is deformed; (7) the contact between the left and right sheet. The above contact pairs are all set to general contact in ABAQUS, and there is friction between the contact surfaces. Since friction has little effect on the calculation results, the friction coefficient is set to a fixed value of K_f_ = 0.2 [15,16,17].

### 2.3. Analysis of Simulation Results

#### 2.3.1. Stress Distribution after Riveting

(1) Influence of rivet spacing on residual stress distribution of sheets

**Figure 7:**
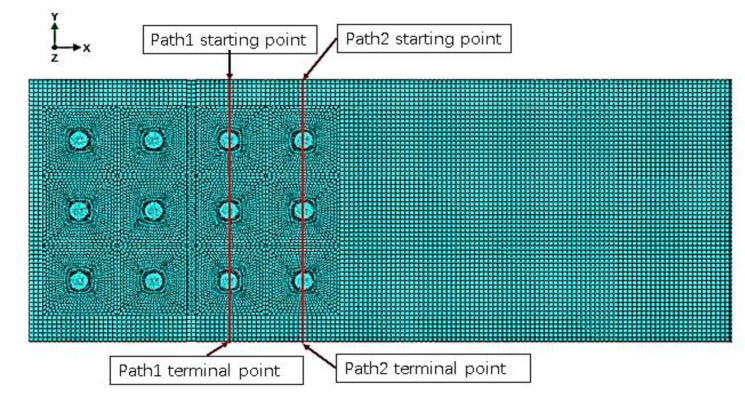
Meshing of model with the strap butt joints.

**Figure 8:**
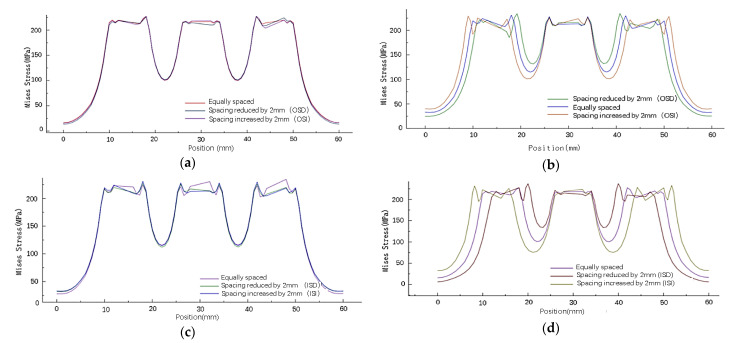
Mises stress distribution around the hole in the wall with different rivet spacing: (**a**) Mises stress distribution around the hole of path1 sheet under changes in the outer rivet spacing; (**b**) Mises stress distribution around the hole of path2 sheet under changes in the outer rivet spacing; (**c**) Mises stress distribution around the hole of path2 sheet under changes in the inner rivet spacing; (**d**) Mises stress distribution around the hole of path1 sheet under changes in the inner rivet spacing.

The stress analysis of the inner (path1) and outer (path2) columns of rivets on the right-side sheet in Figure 7 shows that changing the spacing of the inner or outer rivets will only affect the residual stress around the holes of this column of rivets. As shown in Figure 8, the distribution has almost no obvious influence on the stress around the holes of other columns of rivets. 

**Figure 9:**
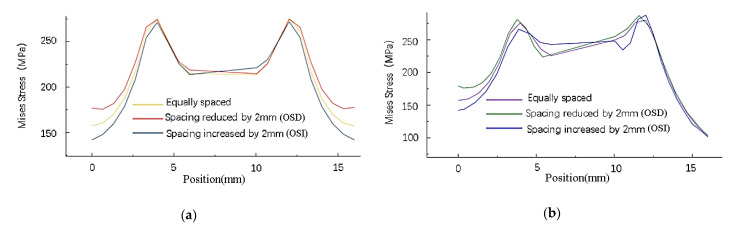

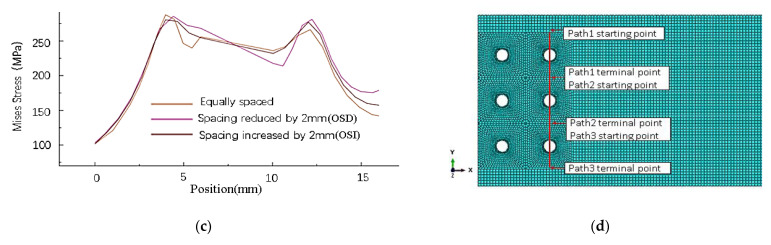
Mises stress distribution around a single rivet hole after changing the rivet spacing: (**a**) Mises stress distribution around No. 1 rivet hole; (**b**) Mises stress distribution around No. 2 rivet hole; (**c**) Mises stress distribution around No. 3 rivet hole; (**d**) Stress path setting around each rivet hole.

As shown in Figure 9, with four times the diameter as the reference distance, when the rivets at both ends move closer to the middle, the maximum stress value around the rivet hole increases, and the minimum stress value between the rivets increases. Conversely, when the rivets at both ends move away from the middle, the maximum stress value around the rivet hole decreases, and the minimum stress value between the rivets decreases. In contrast, the stress distribution around the hole of the middle row of rivets is less affected.

(2) Influence of rivet row spacing on residual stress distribution of sheets

**Figure 10:**
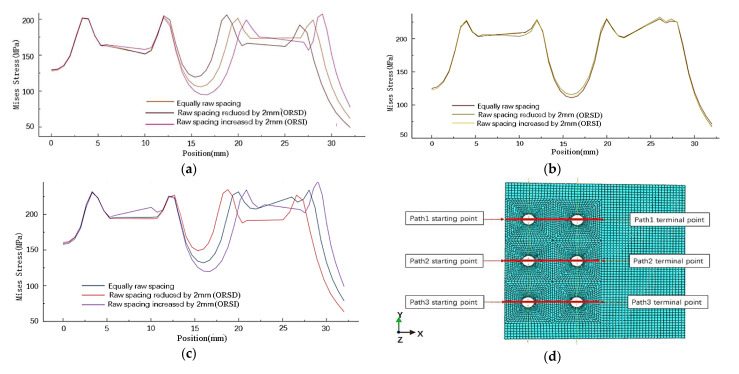
Mises stress distribution in X-direction path of the sheet under different row spacing: (**a**) Mises stress distribution of path1; (**b**) Mises stress distribution of path2; (**c**) Mises stress distribution of path3; (**d**) Path setting of the siding along the stretching direction.

Because the rivet row spacing is changed, the X coordinate of the original rivets in the same column is different. Therefore, the stress distribution of the sheet is studied according to the path in Figure 10d. As shown in Figure 10, changing the row spacing of rivets also has almost no obvious effect on the stress around the holes of other columns of rivets. When the row spacing of the rivets at both ends of one column changes, the stress distribution around the rivet hole is uneven, and the stress distribution of the rivets in the middle row has little influence. When the row spacing of rivets at both ends increases, the maximum stress on the outside of the rivet increases, the maximum stress on the inside decreases, and the minimum stress between the rivets decreases; on the contrary, when the row spacing decreases, the maximum stress on the outside of the rivet decreases, the maximum stress on the inside increases, and the minimum stress between the rivets increases.

Based on the obtained simulated results of the stress distribution of the sheets by changing the spacing and row spacing of the inner and outer rivets, it can be seen that when the position of the outermost rivet of a column of rivets is changed, only the stress distribution of that column of rivets is affected, and the stress value and distribution of other columns of rivets are not affected. When the outermost rivet in the same column moves, it changes the maximum stress around the rivet itself and the stress distribution between adjacent rivets. When the spacing between the rivets is reduced, the maximum stress around the rivet hole increases, and the minimum stress between the rivets increases. On the contrary, when the spacing of the rivets at both ends is increased, the maximum stress around the rivet hole decreases, and the minimum stress between the rivets decreases. Changing the row spacing of the rivets at both ends, the stress distribution perpendicular to the stretching direction hardly changes, while the stress distribution parallel to the stretching direction changes significantly. When the rivet row spacing decreases, the maximum stress around the rivet hole increases, and the minimum stress between the rivets increases. Conversely, when the rivet row spacing at both ends increases, the maximum stress around the rivet hole decreases, and the minimum stress between the rivets decreases. Changing the position of the two columns of rivets inside and outside has roughly the same influence on the size and distribution of the residual stress of the sheet. 

#### 2.3.2. Stress Distribution after Stretching

(1) Influence of rivet spacing change on stress distribution of sheets

**Figure 11:**
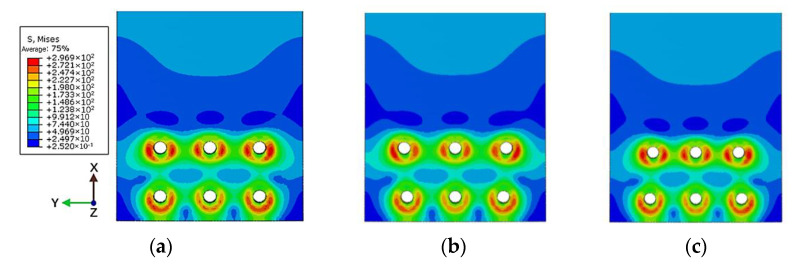
Mises stress distribution of sheet under maximum tensile load: (**a**) spacing is 4 times the diameter; (**b**) increase the spacing by 2 mm (OSI); (**c**) decrease the spacing by 2 mm (OSD).

**Figure 12:**
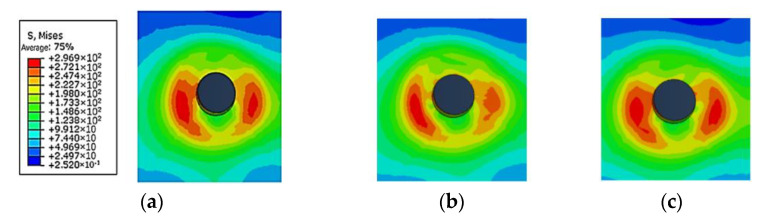
Schematic diagram of Mises stress distribution around the outer No. 1 rivet hole: (**a**) spacing is 4 times the diameter; (**b**) increase the spacing by 2 mm (OSI); (**c**) decrease the spacing by 2 mm (OSD).

Taking the basic connectors with equal spacing and equal row spacing as a comparison, the influence of changing rivet spacing on the stress distribution of the connectors after stretching was studied. As shown in Figure 11 and Figure 12, when the distance between the rivets is 4 times the diameter of the rivet, the stress distribution around the rivet hole is uniform after stretching. When the distance between the outer ends of the rivet increases, the stress on the outside of the hole increases and the stress on the inside decreases. Conversely, when the distance between the rivets at both ends decreases, the stress on the outside of the hole decreases, and the stress on the inside increases. The deviation makes the stress distribution around the hole of the sheet show an irregular trend.

**Figure 13:**
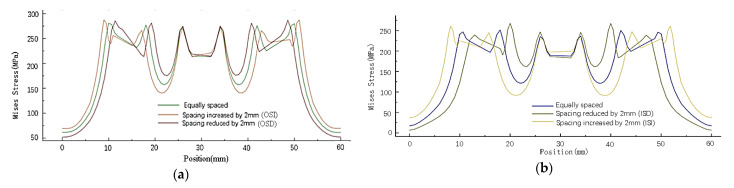
Stress distribution of the connector after stretching under changing the spacing of rivets: (**a**) Stress distribution along path2 after changing the outer rivet spacing; (**b**) stress distribution along path1 after changing the inner rivet spacing.

As shown in Figure 13, the stress distribution of the strap butt joints after tension caused by the change of spacing between the inner and outer rivets is roughly similar. After a tensile load is applied, changing the spacing of the rivets makes the changes in the residual stress around the holes of the rivets at both ends more obvious, while the residual stress around the holes of the middle rivet changes less. When the spacing between the rivets decreases, the residual stress around the holes at both ends increases, the stress inside the hole increases, and the stress outside decreases. When the spacing between the rivets increases, the residual stress around the holes at both ends decreases, the stress outside increases and the stress inside decreases.

(2) Influence of rivet row spacing change on stress distribution of sheets

**Figure 14:**
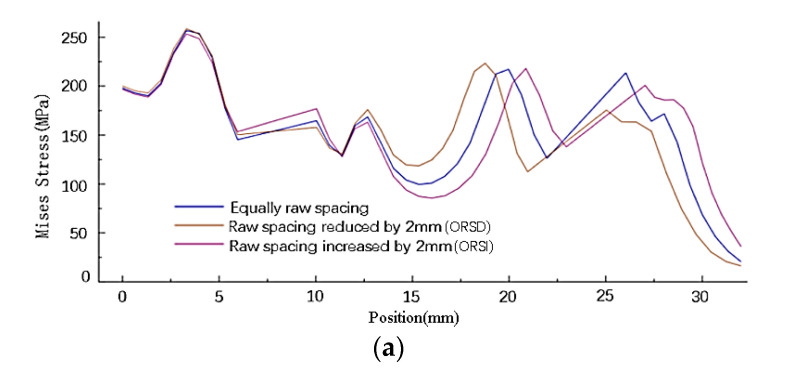

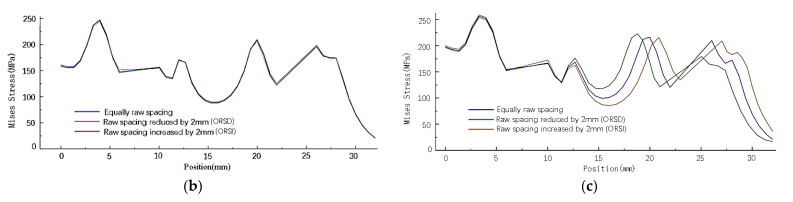
Stress distribution of the connector after stretching under changing the raw spacing of rivets: (**a**) stress distribution along path1; (**b**) stress distribution along path2; (**c**) stress distribution along path3.

The stress distribution of the sheet along the X-direction path is shown in Figure 10d. As shown in Figure 14, the change in the row spacing of rivets changes the stress distribution around the rivet holes and the stress distribution between the rivets. The stress distribution has little influence on those rivets whose raw spacing is not changed. The increase or decrease in the row spacing of the rivets at both ends reduces the residual stress on the side of the sheet near the loading position. When the row spacing of the rivets at both ends increases, the residual stress around the hole decreases, and the minimum stress between the rivets decreases. On the contrary, when the rivet row spacing decreases, the residual stress around the hole increases, and the minimum stress between the rivets increases. The change of stress distribution caused by the change of row spacing of the inner and outer rivets is roughly the same.

#### 2.3.3. Nail Load Ratio

**Figure 15:**
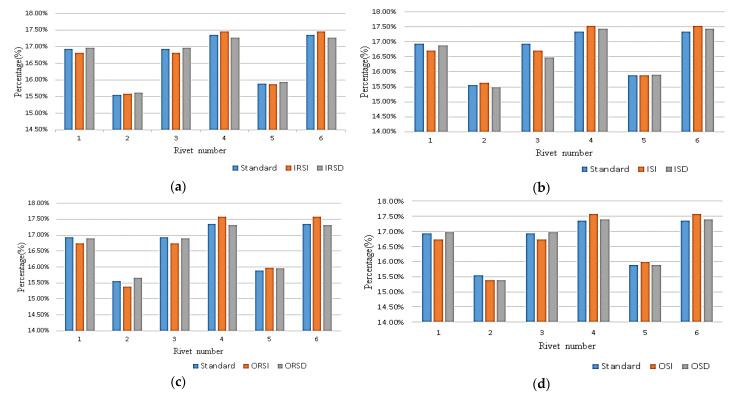
Nail loading ratio of different rivet layouts: (**a**) the nail load ratio after changing the inner row spacing; (**b**) the nail load ratio after changing the inner spacing; (**c**) the nail load ratio after changing the outer row spacing; (**d**) the nail load ratio after changing the outer spacing.

The nail load ratio refers to the percentage of the load borne by each rivet in the strap butt joint to the total applied load. As shown in Figure 15, changing the spacing or row spacing between rivets changes the nail load distribution of each rivet. For the same column of rivets perpendicular to the tensile direction, the rivets at both ends are stressed, the middle rivet bears less force, and the force layout presents a “V” shape; for rivets parallel to the tensile direction, the bearing force of the rivet that is far away from the load application position is large, and the rivet close to the load application position is small, which conforms to the general nail load distribution law. When the distance between rivets is reduced by changing the rivet spacing or row spacing, the nail load at both ends of a column of rivets close to the loading position increases, and the nail load at both ends of a column of rivets far away from the loading position decreases. When the distance between rivets increases, the nail load at both ends of a column of rivets far away from the loading position increases. Meanwhile, the nail load at both ends of a column of rivets close to the loading position decreases. 

Within the specified allowable range (the distance between the rivets in the same column is 3d_0_, d_0_ is the diameter of rivet rod), moving the outer rivets perpendicular to the tensile direction can only reduce the distribution ratio of the nail load as much as possible, but cannot achieve the same distribution ratio of the nail load. 

## 3. Experiments

### 3.1. Preparation of Test Pieces

**Figure 16:**
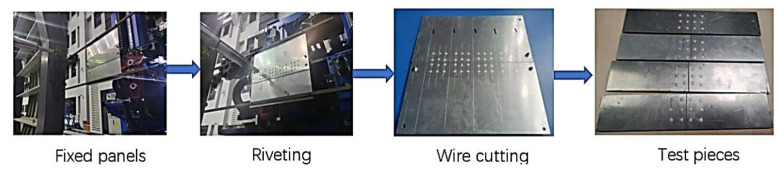
Preparation process of test pieces.

- Experimental equipment and preparation:

The process shown in Figure 16 is used to prepare the test pieces. The riveting experiment equipment adopts the horizontal dual-machine combined automatic drilling and riveting machine, independently developed by Zhejiang University [15]. The end effector of the inner riveting head of the automatic drilling riveting machine is mainly used for clamping and riveting; the end effector of the outer riveting head is mainly used for clamping, hole making, countersinking, nail feeding, nail insertion and riveting. Four laser displacement sensors are installed on the outer riveting head, which can ensure that the normal vector of the end effector is consistent with the normal direction of the hole-making area of the workpiece.

- Clamping and fixing of the test piece:

The test sheet is made according to the size of the test piece, and the plug gauge is used to measure and control the gap between the sheets. The left and right sheets are both 150 mm × 300 mm AL2024-T3 aluminum alloy sheet, and the strap plate is 150 mm × 72 mm AL2024-T3 aluminum alloy sheet. Bolts are used to connect the sheets and strap plate to facilitate the riveting operation. Holes are drilled at the same position of the two plates, and bolts are used to fix the test piece on the positioning fixture. After installation, the test board is fixed in each degree of freedom during riveting, and it is not easy to shake and deform.

- Overall riveting of strip plate connectors:

The riveting drill depth and countersink angle are determined before riveting, trial riveting operations are performed, the head size and head height of the rivet after riveting are measured, and when the rivet size meets the requirements, the order to carry out riveting processing is followed. During the riveting process, the automatic drilling and riveting machine presses the riveted part to ensure the vertical relationship between the test part and the rivet.

- Wire cutting processing:

The test piece after riveting is subjected to wire cutting to obtain the riveting piece required for the test. The structure size of the test piece is consistent with Scheme 1 and Scheme 2 in the finite element simulation.

### 3.2. Quasi-Static Tensile Test of the Strap Butt Joints

**Figure 17:**
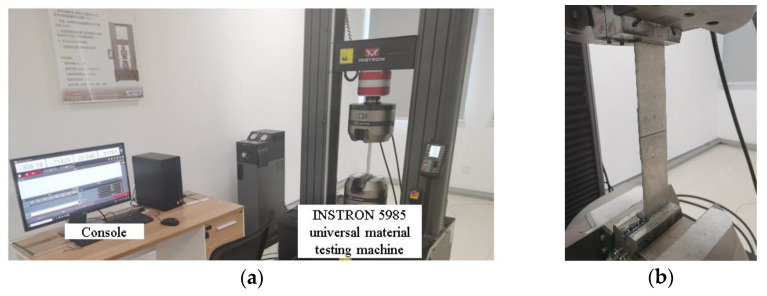
Static tensile test equipment and test scene: (**a**) INSTRON 5985 universal material testing machine; (**b**) schematic diagram of specimen clamping.

The INSTRON 5985 universal material testing machine is selected for static tensile testing equipment with strap butt joints, as shown in Figure 17. The length and width of the test piece is set in the experimental console panel, the tensile rate is set to 1 mm/min and then, the test is started. Three repeated tests are performed on the test pieces under each rivet layout to reduce experimental errors and contingency.

### 3.3. Test Group and Result Analysis

The quasi-static tensile test can accurately and intuitively reflect the connection strength of the joints. By averaging and standard deviation of the connection strengths obtained from three repeated tests under each rivet layout, the test results are shown in Table 2.

**Figure 18:**
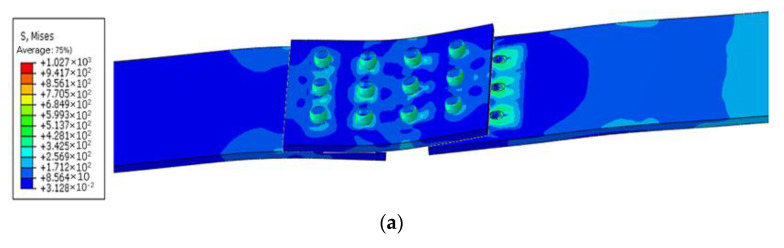

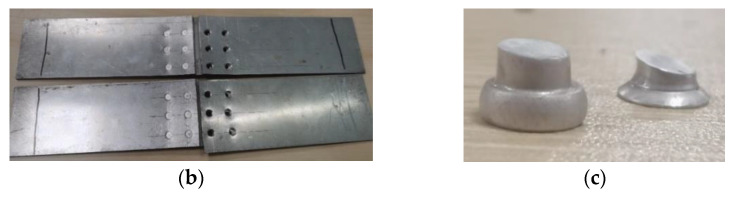
Comparison of failure modes of the simulation and experiment: (**a**) simulation results; (**b**) experimental results; (**c**) rivet shear failure results.

Changing the distance between the internal and external rivets has the same influence on the connection strength. When the rivet spacing increases, the load bearing of the rivets at the outer ends near the loading position decreases, while the uniformity of the external nail load distribution increases, so the connection strength increases. On the contrary, when the rivet spacing at both ends is reduced, the load-bearing capacity of the rivets at the two outer ends close to the loading position increases, so that the bearing load of the rivets at both ends is greater; the uniformity of the outer nail load distribution decreases, so the connection strength decreases. As shown in Figure 18, when the strap butt joints fail, all rivets undergo shear failure, which is consistent with the finite element simulation results. All of the above shows that the finite element model is accurate and reliable.

**Figure 19:**
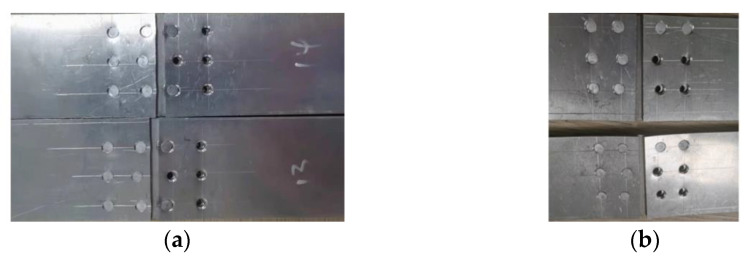
Part of the connector rivets fall off: (**a**) increase the rivet row spacing at both ends of the inner side; (**b**) decrease the rivet row spacing at both ends of the inner side.

The change of the rivet row spacing at both ends of the inner and outer sides has different influences on the strength of the strap butt joints. Since the inner rivets far away from the loading position have a larger nail load than the outer rivets, they are more likely to fail first. As a result, changing the inner row spacing to change the uniformity of the nail load distribution cannot achieve sufficient load-bearing optimization for the rivets with larger loads at both ends. The increase or decrease in the internal row spacing will reduce the connection strength. In the same way, because the external rivets close to the loading position bear a relatively small load, no matter whether the external row spacing increases or decreases, the connection strength increases. When part of the rivets of the strap butt joints breaks, the external force applied by the testing machine undergoes a sudden change. At this time, the testing machine records the data and stops applying the load. Therefore, as shown in Figure 19, an increase or decrease in the rivet row spacing at both ends of the inner side can only make part of rivets break off.

## 4. Conclusions

In this paper, finite element simulations and tensile tests are used to study the influence of the rivet position of the strap butt joints on the connection quality. The influence law of connection strength of the strap butt joints caused by changing the rivet spacing or row spacing with different positions is concluded as follows:

(1) Appropriate adjustment of the position of rivets can change the ratio of the nail load and the stress distribution of the connector. Changing the rivet spacing or row spacing will only affect the stress distribution of one column of rivets at the changed position, and hardly affect the stress distribution of other columns of rivets. When the distance between rivets is decreased by changing the spacing or raw spacing between rivets, the load on both ends of a column of rivets close to the loading position increases, and the load on both ends of a column of rivets far away from the loading position decreases.
(2) The change in the spacing between internal and external rivets has the same influence on the connection strength of the strip plate connector: when the rivet spacing at both ends of one column increases, the connection strength increases. The change of the rivet row spacing at both ends of the inner and outer sides has a different influence on the strength of the strap plate connector: changing the inner row spacing reduces the connection strength, while changing the outer row spacing increases the connection strength. The connection strength can be maximized by reducing the outer row spacing. The average strength is 22,735 N, and the strength is increased by about 3.33%.
(3) In the design of a large riveted connection structure, it is necessary to consider the uneven riveting layout. Shifting the position of the outer rivets inward as a whole or increasing the spacing of each column of rivets, if the design allows, can be considered, so as to improve the overall connection performance.

## Figures and Tables

**Table 1:** Material parameters of connectors.

Material Name	Elastic Modulus (MPa)	Poisson’s Ratio	Yield Strength (Mpa)	Density (Kg/m^3^)	Strength Coefficient C(Mpa)	Hardening Exponent m
AL2117-T4	71,700	0.33	480	2690	544	0.15
AL2024-T3	72,400	0.33	310	2730	745	0.164

**Table 2:** Average value and standard deviation of static tensile strength of different experimental groups.

Group	Moved Rivet Numbers	Moving Direction	Average Failure Load (N)	Standard Deviation
The control group	/	/	22,003	78.407
OSI	1,3,10,12	Increase spacing	22,501	55.154
OSD	1,3,10,12	Decrease spacing	21,220	547.158
ORSI	1,3,10,12	Increase row spacing	22,030	304.85
ORSD	1,3,10,12	Decrease row spacing	22,735	56.062
ISI	4,6,7,9	Increase spacing	22,349	530.073
ISD	4,6,7,9	Decrease spacing	21,915	375.921
IRSI	4,6,7,9	Increase row spacing	21,281	56.149
IRSD	4,6,7,9	Decrease row spacing	21,148	257.416

## Data Availability

The data presented in this study are available on request from the corresponding author.
